# Review on Biological Characteristics of Kv1.3 and Its Role in Liver Diseases

**DOI:** 10.3389/fphar.2021.652508

**Published:** 2021-05-21

**Authors:** Junda Liu, Xiong-Wen Lv, Lei Zhang, Hua Wang, Jun Li, Baoming Wu

**Affiliations:** ^1^ First Affiliated Hospital of Anhui Medical University, Hefei, China; ^2^ School of Pharmacy, Anhui Medical University, Hefei, China; ^3^ Inflammation and Immune Mediated Diseases Laboratory of Anhui Province, Hefei, China

**Keywords:** liver diseases, Kv1.3, margatoxin, macrophages, Kupffer cells

## Abstract

The liver accounts for the largest proportion of macrophages in all solid organs of the human body. Liver macrophages are mainly composed of cytolytic cells inherent in the liver and mononuclear macrophages recruited from the blood. Monocytes recruitment occurs mainly in the context of liver injury and inflammation and can be recruited into the liver and achieve a KC-like phenotype. During the immune response of the liver, macrophages/KC cells release inflammatory cytokines and infiltrate into the liver, which are considered to be the common mechanism of various liver diseases in the early stage. Meanwhile, macrophages/KC cells form an interaction network with other liver cells, which can affect the occurrence and progression of liver diseases. From the perspective of liver disease treatment, knowing the full spectrum of macrophage activation, the underlying molecular mechanisms, and their implication in either promoting liver disease progression or repairing injured liver tissue is highly relevant from a therapeutic point of view. Kv1.3 is a subtype of the voltage-dependent potassium channel, whose function is closely related to the regulation of immune cell function. At present, there are few studies on the relationship between Kv1.3 and liver diseases, and the application of its blockers as a potential treatment for liver diseases has not been reported. This manuscript reviewed the physiological characteristics of Kv1.3, the relationship between Kv1.3 and cell proliferation and apoptosis, and the role of Kv1.3 in a variety of liver diseases, so as to provide new ideas and strategies for the prevention and treatment of liver diseases. In short, by understanding the role of Kv1.3 in regulating the functions of immune cells such as macrophages, selective blockers of Kv1.3 or compounds with similar functions can be applied to alleviate the progression of liver diseases and provide new ideas for the prevention and treatment of liver diseases.

## Introduction

Liver diseases, including hepatitis B virus (HBV) and hepatitis C virus (HCV) infection, alcoholic liver disease (ALD), non-alcoholic fatty liver disease (NAFLD) and related cirrhosis, liver failure (LF), and hepatocellular carcinoma (HCC), are the main causes of disease and death in the world. In China, viral hepatitis, non-alcoholic fatty liver disease (NAFLD), and alcoholic liver disease (ALD) affect about 300 million people, 130 million of whom are affected by ALD and NAFLD, accounting for half of the population with ALD and NAFLD in the world. Since 1992, the number of new cases of HBV infection has dropped dramatically as a result of expanded immunization, while the number of patients with alcoholic and non-alcoholic fatty liver disease, which causes fibrosis and eventually cirrhosis and even liver cancer, is increasing at an alarming rate. Based on the latest GLOBOCAN data, liver cancer is the second most common cancer in China, it killed 391,152 people in 2020, accounting for 47% of the global total death by liver cancer (World Health Organization, 2020). Here, we summarized the physiological characteristics of Kv1.3, the relationship between Kv1.3 and cell proliferation and apoptosis, and the role of Kv1.3 in several liver diseases, so as to provide new ideas and strategies for the prevention and treatment of liver diseases.

## Physiological Function and Biological Characteristics of Kv1.3

**FIGURE 1:**
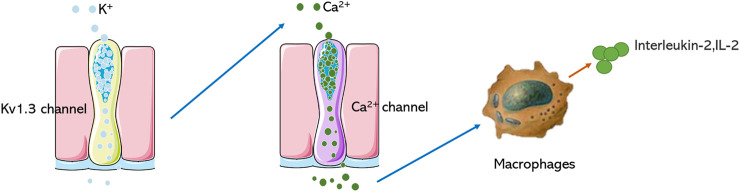
Mechanism of Kv1.3 in regulating immune cells. Blocking the Kv1.3 channel prevents the opening of the Ca2+ channel of macrophages and reduces the secretion of IL-2.

The voltage-dependent potassium ion channel (Kv) belongs to a large and very diverse family, consisting of multiple subfamilies (Kv1-4). Kv plays an important role in the physiological and pathophysiological processes of excitatory and non-excitatory cells, is involved in regulating Ca^2+^ signal, cell volume, secreted cytokines, cell proliferation, and migration (Serrano-Novillo et al., 2019; Wulff et al., 2009). Kv1.1-1.7 have been studied extensively, Kv1.3 belongs to the subtype of voltage-gated potassium channels, the protein is made up of 575 amino acids. It has six transmembrane segments (S1-S6), including a voltage sensor (S1-S4), which is a detector of the membrane voltage change, a pore‐forming domain (S5-S6), which is highly selective for‐potassium flowing (Pérez-Verdaguer et al., 2016), when membrane potential changes, the positive charges in the S4 domain will translocate and lead to conformational change, resulting in opening or closing of the pore (Hendrickx et al., 2020). The K^+^ channel predominantly regulates membrane potential, efflux of K^+^ of ions through the pore can evoke Ca^2+^ influx and regulate the function of many enzymes and transcription factors, which induces a cascade of pharmacological reactions (Abe et al., 2019). Kv1.3 is a crucial regulatory protein in the immune response, which was initially discovered in T lymphocytes. It is an initiating factor in the regulation of T cell activity and differentiation, and its function is closely related to immune regulation (Feske et al., 2015; Jaimes-Hoy et al., 2017; Tschritter et al., 2006). Blocking Kv1.3 can inhibit Ca^2+^ signal transduction, T cell proliferation, and IL-2 secretion (Decoursey et al., 1984; Garcia et al., 2018) (Figure 1), which could obviously alleviate immune reaction by suppressing cytokines secretion. Many studies have shown that besides being highly expressed in T lymphocytes, Kv1.3 is also expressed in other immune cells, including microglia, lymphocytes, and macrophages. Using an electrophysiological technique, characteristic currents of Kv1.3 were recorded in human bone marrow‐derived macrophages, mammalian macrophages, microglia, monocytes, human alveolar macrophages, and other cells (Gallin, 1984; Mckinney and Gallin, 1990; Nelson et al., 1990; Kotecha and Schlichter, 1999; Cahalan et al., 2001; Mackenzie et al., 2003; Xie et al., 2015; Lowinus, 2020; Wang, 2020).

## Kv1.3 Channel Blockers

The Kv1.3 channel shows an unusual characteristic compared with other potassium channel subtypes, this channel is more sensitive to toxin blockers that possess a structurally diverse channel-interacting interface, such as the scorpion toxin autoimmune drug Wenxin group which binds the anti-parallel β-sheet domain, the scorpion toxin *Buthus martensi* mainly leverages the turn motif between the a-helix and antiparallel β*-*sheet domains. These unique pharmacologic characteristics could make it easy to discover novel blockers of the Kv1.3 channel. Interestingly, blocking the Kv1.3 channel can alleviate or treat diverse diseases, and different Kv1.3 blockers have great clinical significance in pharmacological regulation of Kv1.3 activity. Kv1.3 channel blockers include tetramethylamine (TEA), 4-aminopyridine (4-AP), quinine, verapamil, diltiazem, cetiedil, trifluoperazine, chlorpromazine, and other Kv channel blockers with different chemical properties. These blockers can inhibit cellular activities and gene expression, as well as the secretion and proliferation of lymphocytes. The ability of Kv channel blockers to inhibit cell proliferation has been found in a variety of cell types except the immune system (Cidad et al., 2020). At present, there are many synthetic inhibitors of Kv channels. However, scorpion venom which contains short peptides (20–80 amino acid residues) is the main source of Kv1.3 channel inhibitors (Shen et al., 2017). More and more selective Kv1.3 blockers are being used in studies, such as Vm24 (Cidad et al., 2020), SHK-170 (Varga et al., 2012), Mokatoxin-1 (Beeton et al., 2011), and OSK1-20 (Takacs et al., 2009) Margatoxin (MgTX) is considered a high-affinity and selective inhibitor of the Kv1.3 channel (Jang et al., 2011; Anangi et al., 2012; Tucker et al., 2013; Bhuyan and Seal, 2015). However, MgTX has high homology with other scorpion peptide sequences and has high affinity to both Kv1.3 and Kv1.2 channels (Bartok et al., 2014), suggesting that the specific selectivity of MgTX to Kv1.3 should be further studied. Due to their selectivity, potency, and stability, venom-derived ion channel inhibitors have become proper candidate molecules for research. As an example, MgTX is currently considered as a stable inhibitor of Kv1.3 (Knaus et al., 1995; Spencer et al., 1997; Ghanshani et al., 2000; Menteyne et al., 2009; Mast and Fadool, 2012; Schwartz et al., 2017). However, MgTX has some disadvantages in clinical application. Because it is a kind of polypeptide and a large molecule, it has many limitations on the dosing methods. There may be rejection by patients or doctors because it is a scorpion venom. Also, MgTX may have some affinity to other Kv channels. It is therefore important to find molecule compounds with higher selectivity to Kv1.3 than MgTX.

## Kv1.3 and Proliferation

Research on the function of Kv1.3 mainly focuses on the role of Kv1.3 in immune system diseases, however, the role of Kv1.3 in cell proliferation is also related to its regulation of immune function. It was first reported in 1984 that Kv1.3 is related to cell proliferation, and there are three mechanisms to regulate cell proliferation according to different cell types. The first mechanism is called the “membrane potential model” (Tschritter et al., 2006; Chandy and Norton, 2017). When the membrane potential is hyperpolarized in T lymphocytes, which causes K^+^ to flow from the cell to the extracellular, it provides the driving force for Ca^2+^ to flow into the cell and further promotes the activation of Ca^2+^‐dependent transcription factors, leading to cell proliferation. The second mechanism is known as the “voltage sensor model” (Cidad et al., 2012; Jiménez-Pérez et al., 2016), which has been reported in vascular smooth muscle cells. According to this model, Kv1.3 channels, as membrane voltage sensors, are very sensitive to the voltage change of the cell membrane. Membrane depolarization causes channels to open, which promotes the intracellular phosphorylation of tyrosine and serine residues by protein kinase in the MEK-ERK signaling pathway, so as to promote cell proliferation. Macrophages are different from T lymphocytes in that Kv1.3 is located on the T lymphocyte membrane alone, while Kv1.3 and Kv1.5 form a heterotetramer on the macrophage membrane. This form is also shown in glial cells. Cell proliferation is regulated by this special model, known as the “channel balance model” (Vicente et al., 2006; Cidad et al., 2012). Proliferation of glial cells is determined by the ratio of Kv1.3 and Kv1.5. Therefore, Kv1.3 can participate in cell proliferation by regulating intracellular Ca^2+^ as the second messenger, promoting the activation of intracellular transfer factors, or regulating the MEK-ERK signaling pathway to promote cell proliferation, and can also participate in cell proliferation by up regulating Kv1.3 and down regulating Kv1.5 channel expression.

## Kv1.3 and Apoptosis

As we know, T cell activation and proliferation are initially regulated by Kv1.3 on the cell membrane. However, Kv1.3 was also found in the inner membrane of mitochondria, and many studies reported that Kv1.3 in mitochondria was closely related to apoptosis. For example, Kv1.3 on the mitochondrial membrane is involved in the death of CTLl-2 cell lines. In this mechanism, Kv1.3 on the mitochondrial membrane interacts with Bax (Bcl-2 family), triggering apoptosis in the way of toxoid (Pérez-García et al., 2018). PAPTP and PCARBTP are derived from potassium channel inhibitors PAP-1, which have high affinity to Kv1.3 in mitochondria. Application of mitochondrial Kv1.3 channel inhibitors (PAPTP and PCARBTP) can induce glioblastoma cell apoptosis, however, application of a nonspecific mitochondrial Kv1.3 channel inhibitor (Psora-4, PAP-1) in glioblastoma cells only produced a low percentage of apoptosis at ∼30%, because of their low affinity for mitochondria (Szabo et al., 2008; Venturini et al. (2017)). In addition, it is well known that cancer cells may become resistant to apoptosis, and the expression of the Kv1.3 channel may be one of the ways in which these cells are resensitized to apoptotic stimuli. It has been shown that the mouse cytotoxic T lymphocyte cell line CTLl-2 with a Kv1.3 defect is resistant to mitochondria-mediated apoptosis. Kv1.3 expression by transfection resensitized these cells to the apoptosis signal and promoted the apoptosis of CTLL-2 cells (Gulbins et al., 2010). However, not all Kv1.3 channel inhibitors can induce apoptosis of tumor cells. In the cell model of tumor apoptosis simulated by MgTX, it was shown that MgTX could not block the Kv1.3 channel of mitochondria in the cells, because MgTX could not enter through the cell membrane and act on mitochondria (Teisseyre et al., 2019). Therefore, the discovery of small molecule inhibitors with membrane permeability and selective inhibition of the mitochondrial Kv1.3 channel is of great significance, especially for targeting apoptosis and improving the targeted selectivity of compounds.

## Kv1.3 and Acute Liver Injury

Acute liver injury (ALI) involves severe liver injury with abnormal function of liver cells, leading to different clinical syndromes such as clotting disorder, encephalopathy, and circulatory dysfunction. ALI is associated with a high mortality rate from liver diseases, ranging from 30 to 80% (Bernal et al., 2010). Kupffer cells (KCs) are key immune cells in the innate immune system of the liver and play a key role in lipopolysaccharide (LPS)-induced responses, which promote the secretion of inflammatory cytokines, including interleukin-1 (IL-1), interleukin-6 (IL-6), monocyte chemical attractant protein 1 (MCP-1), and tumor necrosis factor-*α* (TNF-*α*). These pro-inflammatory cytokines induce liver cell death, ultimately leading to ALF (Wang et al., 2016). In liver disease, there are two types of macrophages that function as immune cells: resident macrophages and circulating macrophages, which are different from monocytes. Circulating monocytes play an important complementary role in the homeostatic stability of the liver macrophage pool. Liver metabolism or toxic injury leads to the infiltration of a large number of monocyte‐derived macrophages into the liver (Tacke and Zimmermann, 2014). Therefore, monocyte infiltration may be a key event in the development of liver fibrosis. Lipopolysaccharide (LPS) can directly bind to hepatic macrophages toll-like receptor 4 (TLR4), activate the inflammatory response signaling pathway, and produce a large number of inflammatory factors, such as tumor necrosis factor TNF-*α* and interleukin-6 (IL-6), thus causing damage to liver cells. Our research group established two models of liver injury, namely acute liver injury induced by LPS or LPS+ D-gain (galactosamine). The results showed that MgTX, which is one specific blocker of Kv1.3, could reduce the serum levels of TNF-*α*, IL-6, ALT, and AST, reduce the proportion of peripheral mononuclear macrophages CCR^2+^/Gr1^+^ double-positive cell population and IBA-1^+^/CLEC-4F^+^ positive cell expression, reduce the infiltration of peripheral mononuclear macrophages into the liver, and significantly protect liver from acute liver injury (unpublished). Kv1.3 is essential for mononuclear cell migration, and data from some related studies support that Kv1.3 blockers can reduce macrophage migration. Inhibition of Kv1.3 can block chemotaxis of monocytes and infiltration of monocytes into the damaged brain, and reduce release of neurotoxic factors by activated microglia, including reactive oxygen species and pro-inflammatory cytokines, to alleviate brain damage (Eder, 2010; Liu et al. (2012)).

## Kv1.3 and Alcoholic Liver Disease

Throughout the world, alcohol consumption is the leading cause of liver diseases and alcoholism, and liver diseases are the leading alcohol-related chronic diseases (European Association for the Study of Liver, 2012; Rehm et al., 2013). Alcoholic liver diseases include a variety of histopathological changes, ranging from steatosis to alcoholic steatohepatitis, which can result in fibrosis, cirrhosis, and even hepatocellular carcinoma (O’Shea et al., 2010). Alcohol intake promotes the accumulation of acetaldehyde and other reactive oxygen molecules in the liver, producing oxidative stress and leading to impaired hepatocyte metabolism and cell death (Teschke, 2018). Alcohol intake also promotes the growth of Gram‐negative intestinal bacteria, increases intestinal permeability, and improves the level of endotoxin lipopolysaccharides. Excessive lipopolysaccharides in peripheral blood reaches the liver, which can activate Kupffer cells, thereby producing free radicals and inflammatory cytokines, leading to necrotic inflammation and liver fibrosis (Szabo, 2015) Lipopolysaccharides also activate stationary hepatic stellate cells, secrete pro-inflammatory cytokines, and initiate the progression of liver fibrosis (Mandrekar and Szabo, 2009). While it is known that alcohol consumption can trigger an immune response in the liver, the mechanism of this disease status is still not clear. Recent studies have shown that toll-like receptors play a key role in the immune response and alcohol intake-induced upregulation of pro-inflammatory cytokines such as TNF-, IL-1, and the monocyte chemoattractant protein MCP-1. In addition, these immune responses lead to the production of reactive oxygen species, epigenetic changes, and infiltration of monocytes or neutrophils (Gao et al., 2011; Seki and Schnabl, 2012; Wang et al., 2012; Petrasek et al., 2013). Kupffer cells and macrophages are the earliest immune cells to respond to the liver response caused by alcohol. Therefore, macrophages/Kupffer cells play an important role in the pathogenesis of alcoholic liver diseases, and inhibiting inflammatory cytokines, chemokines, and macrophage infiltration is a feasible strategy for the prevention and treatment of alcoholic liver diseases. In our study, we found that MgTX can reduce the secretion of macrophage inflammatory factors (TNF-, IL-1, IL-20) and the infiltration of LY6C^hi^ macrophages into the liver (unpublished). Therefore, Kv1.3 can regulate macrophage function and may be a new target for the prevention and treatment of alcoholic liver disease.

## Kv1.3 and Hepatic Fibrosis

Hepatic fibrosis is related to inflammatory immune response and fibrogenic cytokines. Recent studies have found that Kv1.3 plays an important role in regulating immune cell function and is closely related to a variety of fibrosis diseases. In cardiovascular diseases, fibroblast proliferation is an important feature of the development of heart failure. Protective effects of regulatory T cells (Tregs) on myocardial fibrosis have been previously reported, however, in the late stage of congestive heart failure (CHF), regulatory T cells secrete the fibrogenic cytokine TGF-β to promote myocardial fibrosis. The high affinity of Eplerenone to the Kv1.3 channel enables it to directly antagonize the Kv1.3 channel, inhibit the proliferation of Tregs, and possibly play an immunoregulatory role in the process of CHF (Shao et al., 2018). For respiratory diseases, such as chronic obstructive pulmonary disease, asthma, bronchiolitis (DPB), and cystic fibrosis, the activity of T lymphocytes and their proliferation in surrounding organs depend largely on the activity and expression level of Kv1.3 channels. Therefore, in these acute or chronic respiratory diseases, these channels are also likely to be overactivated or overexpressed in T lymphocytes. Studies have demonstrated that targeting Kv1.3 channels can treat chronic inflammatory diseases, including respiratory diseases such as asthma or lung cancer (Kazama and Tamada, 2016). In addition, chronic inflammation in silicosis may develop into chronic fibrosis in which the extracellular matrix is replaced by fibroblasts and abnormal collagen. The causative factor is that macrophages phagocytose these harmful particles and are activated to release large quantities of mediators, including histamine and serotonin, leading to a fibrotic response. Nicorandil, which is one of the potassium channel blockers, can improve pulmonary fibrosis by regulating ATP-dependent potassium channels (Tanabe et al., 2012). In a recent study, severe glomerulosclerosis manifested in damaged kidneys, and with the increase of serum creatinine, the kidneys of subtotal nephrectomy rats also displayed diffuse interstitial fibrosis with leukocyte infiltration. Continuous inhibition of the lymphocyte Kv1.3 channel can improve the progression of renal fibrosis by inhibiting renal lymphocyte proliferation (Pinzani, 2008; Nakamura, 2015; Hanson et al., 2018). Hepatic stellate cells (HSCs) are located around hepatic sinuses, accounting for about 5–8% of the total number of hepatocytes. Currently, HSCs are considered one of the key cell types involved in the process of liver fibrosis and related pathophysiological and clinical complications. Activated HSCs produce smooth muscle actin (*α*-SMA), which enables myofibroblasts to proliferate, in addition to promoting the deposition of the extracellular matrix (ECM) and the formation of scar tissue in liver. Activation of myofibroblasts can regulate the expression of pro-fibrogenic factors, such as transforming growth factor-β (TGF-β), thereby promoting the development of fibrosis (Breitkopf et al., 2005; Schachtrup et al., 2011; Gandhi, 2017; Mu et al., 2018). The study found that TGF-β is a key cytokine in liver fibrosis and primary microglia, and that stimulation with TGF-β can increase the amplitude of outward K^+^ current. In mouse brain nerve cell line C8-B4, TGF-β (10 ng/ml) treatment induced a more than two-fold increase in the amplitude of K^+^ current, which was reduced by MgTX (100 nM) (Nörenberg et al., 1994; Fischer et al., 1995; Moussaud et al., 2009). Therefore, Kv1.3 channel inhibitors can regulate the expression of cytokine TGF-β, which is a key factor causing liver fibrosis, suggesting that Kv1.3 channels may alleviate the development of liver fibrosis by reducing the expression of TGF-β. In addition, we administered an interperitoneal injection of MgTX in mice with liver fibrosis to explore the protective effects of Kv1.3 on liver fibrosis through modulating macrophage function (Wu et al., 2020a; Wu et al., 2020b).

## Kv1.3 and Liver Cancer

Numerous studies have shown that cytotoxic CD8^+^ (killing tumor cells) and CD4^+^ (helper T cells promote tumor cell death and/or regulatory T cells) lymphocytes influence the prognosis and response to tumor therapy, and that the functional status of CD8^+^ and CD4^+^ determines the ability to kill tumor cells (Galon et al., 2006; Pagès et al., 2009; Wallis et al., 2015). The function of T lymphocytes is dependent on Ca^2+^ signaling, which is controlled by multiple ion channels to regulate the influx of Ca^2+^ into T cells. In particular, the Kv1.3 channel activates the Ca^2+^ channel through Ca^2+^ release (CRAC) to regulate the membrane potential of human T lymphocytes, providing an electrochemical driving force for Ca^2+^ influx and causing downstream cascade reactions (Panyi et al., 2004; Cahalan and Chandy, 2009; Feske et al., 2012; Hu et al., 2013). There is clear evidence of the importance of Kv1.3 and CRAC channels in T cell activation. Blocking Kv1.3 and CRAC channels inhibits cytokines production and proliferation (Cahalan and Chandy, 2009; Feske et al., 2012; Hu et al., 2013; Although the function of Kv1.3 and CRAC channels in T cell function is relatively clear, their function in cancer remains unclear. It was found that the expression of Kv1.3 in CD8^+^ lymphocytes of head and neck cancer was significantly lower than that of CD8^+^ lymphocytes of normal peripheral blood, suggesting that the expression of Kv1.3 plays an important role in the function of CD8^+^ lymphocytes (Chimote et al., 2017). The change of Kv1.3 expression is closely related to the occurrence and progression of different tumors. Kv1.3 expression varies in different tumors, even at different stages of tumor progression. Previously, it was discussed that Kv1.3 on a cell membrane is involved in cell proliferation, and the Kv1.3 channel on mitochondria is involved in the regulation of cell apoptosis, among which Kv1.3 on mitochondria is considered as a new tumor marker, and decreased Kv1.3 expression on mitochondria can induce the apoptosis of tumor cells (Checchetto et al., 2019). PAPTP and PCARBTP, specific mitochondria inhibitors on Kv1.3, reduced melanoma volume by 90 and 60%, respectively, with no effect on normal organs (Leanza et al., 2017). There have been few studies on the function of Kv1.3 in liver cancer. Yuki Moritoki reported that in patients with primary biliary cirrhosis (PBC), the level of polyclonal IgM produced by peripheral blood mononuclear cells (PBMCs) was significantly higher than that of the CpG exposed control group. In addition, abnormally high levels of anti-mitochondrial antibodies (AMAs) are prevalent and present in PBC patients. The use of Kv1.3 blocker SK can regulate the hyperreactivity of B lymphocytes and inhibit the secretion of AMA, which may be an effective method for PBC treatment (Moritoki et al., 2007). Therefore, Kv1.3 can increase its killing effect by regulating T lymphocyte activity or inducing the apoptosis of liver tumor cells. Reducing the secretion of AMA by a Kv1.3 blocker may achieve the purpose of treating liver cancer.

## Conclusion

**FIGURE 2:**
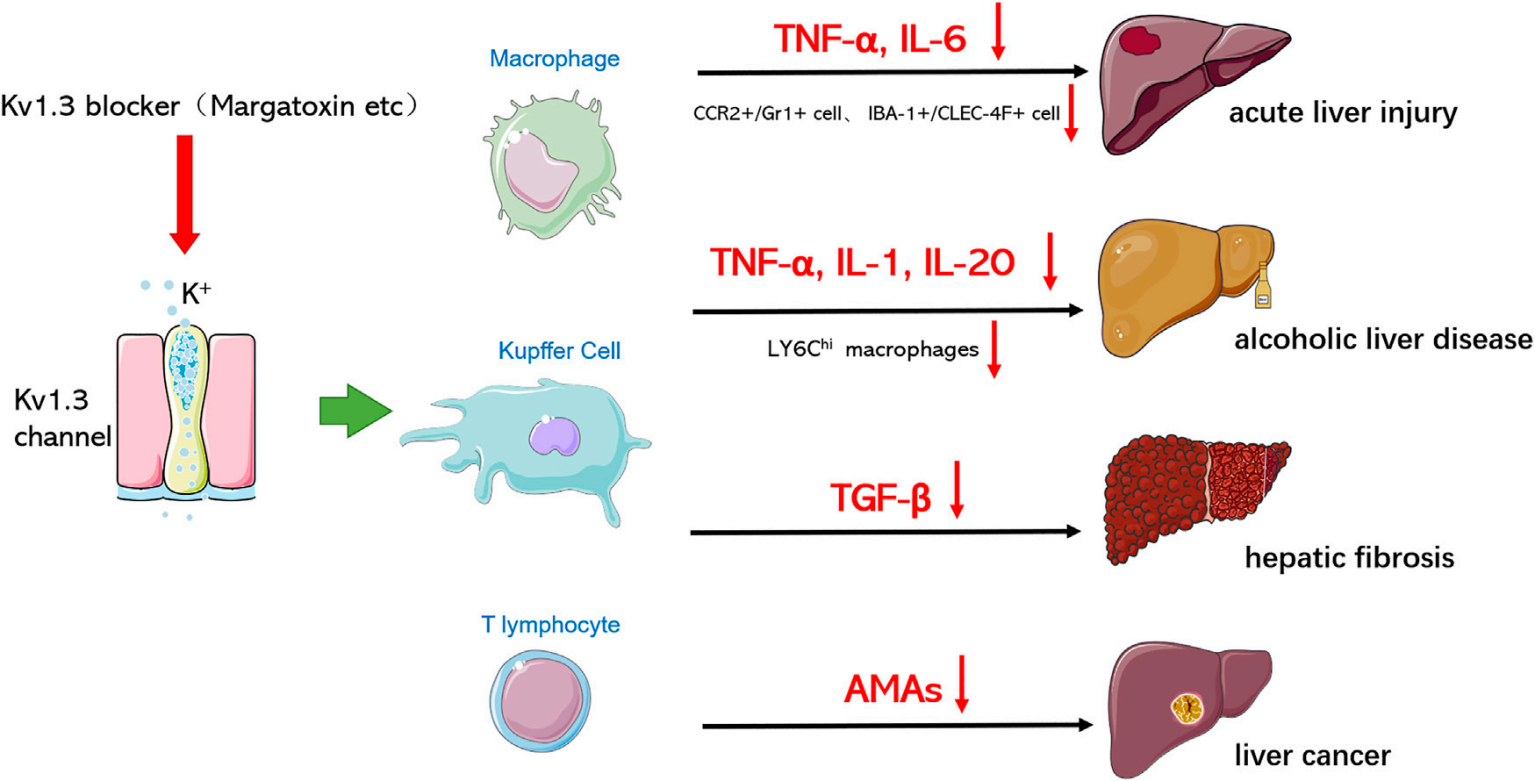
Kv1.3 is involved in various hepatic diseases. Through blocking Kv1.3, Kv 1.3 blockers hinder the progression of various hepatic diseases and reduce damage by influencing macrophages/Kupffer cells and T lymphocytes to secrete cytokines or regulating the polarization of macrophages.

With the deepening of studies on liver diseases, the immune response of the liver is a key factor for the progression of liver diseases, and the regulation of immune cell function is considered as an important strategy for the prevention and treatment of liver diseases. Macrophages/Kupffer cells play an important role in the occurrence and development of liver diseases. For example, macrophages can release a large number of pro-inflammatory cytokines and chemokines to promote the inflammatory response in the liver; on the other hand, macrophages show significant plasticity and can differentiate into different phenotypes with diverse functions, such as M1 and M2 phenotypes, which play different functions in the occurrence and development of liver diseases. We summarized the role and function of Kv1.3 in various hepatic diseases (Figure 2). Therefore, targeting the regulatory effect of Kv1.3 on macrophage recruitment and cytokine secretion may become an effective new strategy for liver diseases treatment in the future.
